# Bilateral congenital upper eyelid eversion: the clinical course and outcome of conservative management

**DOI:** 10.11604/pamj.2014.17.215.3967

**Published:** 2014-03-18

**Authors:** Waheed Ademola Ibraheem

**Affiliations:** 1 Department of Ophthalmology, Ladoke Akintola University Teaching Hospital, Ogbomoso, Oyo State, Nigeria

**Keywords:** Upper eyelid eversion, conservative management

## Abstract

A case of bilateral congenital upper eyelid eversion (CUEE) in an otherwise normal healthy 6 day old neonate of African descent (Nigeria). Pregnancy and delivery history were uneventful. The baby recovered completely 5 days after the commencement of conservative management. This case further gives credence to the usefulness of conservative therapy in the management of CUEE.

## Introduction

Congenital upper eyelid eversion (CUEE) is a rare abnormality of unknown etiology. It could be a potential threat to visual maturation causing amblyopia if not managed promptly and appropriately. Congenital upper eyelid eversion (CUEE) first came into the domain of modern medicine in 1896 when it was reported by Adams as a case of double congenital ectropion [1]. It has since then being recognized as a clinical entity. In Nigeria, 6 cases have been so far reported [2–5]. CUEE is a rare disorder of unknown etiology which sufferers are usually free of other abnormalities (ocular/ systemic). The prevalence of CUEE is skewed positively in favor of neonates with Down syndrome, colloidal skin and those from black race as it is seen in our patient [6–9].

CUEE is commonly bilateral though, may be asymmetrical as in this index patient, unilateral cases have also been previously reported [6]. The disorder is characterized by prolapse of edematous conjunctivae from everted eyelids usually at birth. However, late presentations do exist in the literature [8, 10].

Usually, CUEE do resolves with no ocular sequelae when promptly and appropriately managed otherwise it could be complicated with stimulus deprivation amblyopia [11]. Additionally, a case of CUEE complicated with cornea perforation was once reported in the literature in a 7 month old infant [6].

Although, the exact aetiopathogenesis of CUEE is not known, several theories have been advanced. Among these are: orbicularis hypotonia [12]; birth trauma; vertical shortening of the anterior lamellar or vertical elongation of the posterior lamellar of the eyelid; failure of the orbital septum to fuse with the levator aponeurosis (with adipose tissue interposition) [13]. Additionally, when present, orbicularis spasm could further worsen the chemosis. This it does by causing a vicious cycle of vicious cycle of conjunctival strangulation and chemosis secondary to venous stasis [14].

## Patient and observation

We report a case A 6 day old female neonate was referred to our center on account of bilateral eversion of the upper eyelids since birth. The baby was a full term product of an uneventful pregnancy delivered via spontaneous vaginal route. There was no use of instrumentation and she cried spontaneously at birth.

Further information gathered from the referral note revealed that the child has been on gentamycin eye drop and systemic antibiotics but with no apparent improvement 6 days prior to presentation- the reason for referral.

**Figure 1:**
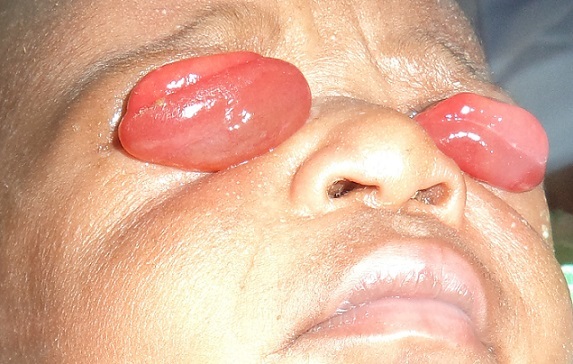
Picture showing the child with bilateral upper eyelid eversion and prolapsed chemotic conjunctiva

**Figure 2:**
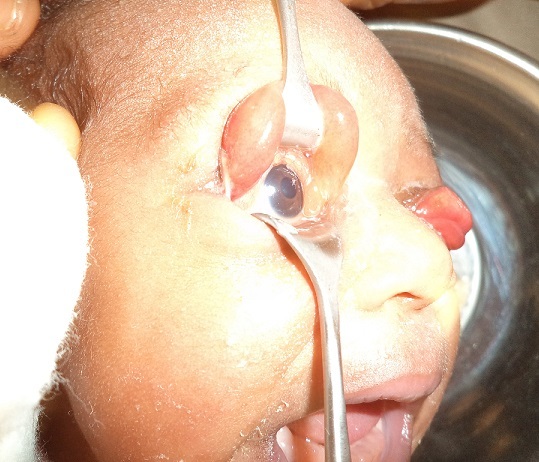
Picture showing normal right globe

**Figure 3:**
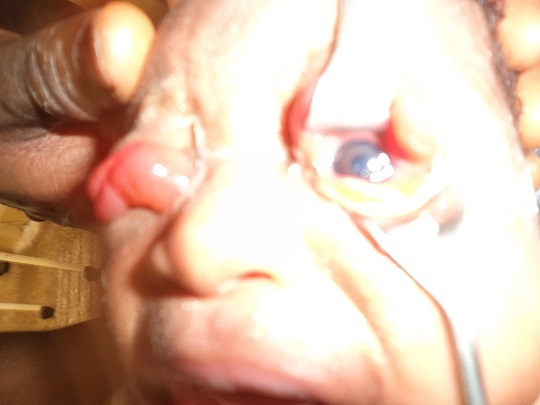
Picture showing normal left eyeball

Examination at presentation revealed an otherwise normal child with bilateral complete eversion of the upper eyelids. There were marked chemosis bilaterally worst in the right (Figure 1). Both eyes were double-everted using lid retractor in order to assess the conditions of the two eyeballs which were both found to be normal (Figure 2, Figure 3). Attempt at repositioning the eyelids was unsuccessful because of the marked chemosis.

**Figure 4:**
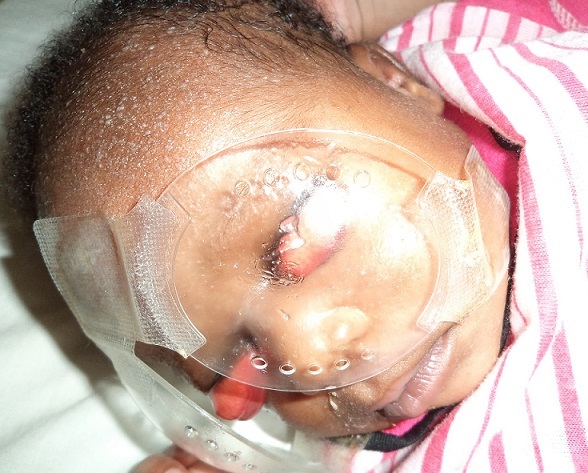
Picture showing the child with perforated transparent catellar shield

The child was admitted into Special baby care unit (SBCU) of our hospital to facilitate close monitoring. She was also reviewed by the pediatrician and no other abnormality was found. Perforated transparent catellar shield was applied over the eyes bilaterally to prevent trauma to the conjunctivae (Figure 4). She was commenced on hypertonic saline 4 hourly and piece of gauze soaked with hypertonic saline was placed over the prolapsed chemosed palpebral conjunctivae for 3hours once in a day. She was also commenced on 2hourly ciprofloxacin hydrochloride USP equivalent to ciprofloxacin 0.3% w/v) and maxitol ointment at night.

**Figure 5:**
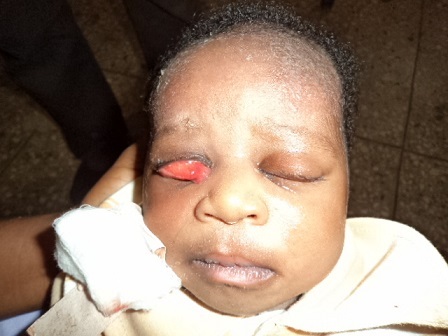
Picture showing left reverted upper eyelid and right resolving chemosis

On the 3rd day of admission, the chemosis on the right had resolved significantly but there was still poor right lid opening (Figure 5). However, on the 5th day of admission, both chemosis had resolved totally with spontaneous eye lid opening. Both eyeballs were normal.

## Discussion

Generally, conservative management is favoured and surgical options usually become necessary in recalcitrant type [15–17]. Conservative treatment is mostly supportive and it includes the use of 5% hypertonic saline and lubricant [3]. The advantage of this approach is that it is non-invasive. Documented surgical options include the use of compression sutures [4], tarsorrhaphy with excision of redundant conjunctiva, subconjunctival injection of hyaluronic acid, fornix sutures, and full thickness skin graft to the upper lid [6].

In the index case, our goal of management was to prevent secondary bacterial infection and enhance speedy reabsorption of the conjunctival oedema. This we achieved through conservative approach with the use of catellar shield, topical hypertonic saline, prophylactic topical antibiotics and maxitol ointment.

In conclusion, we advocate for the creation of awareness among healthcare professionals both in public and private facilities particularly in obstetric and neonatal care of the existence of this potentially sight threatening congenital anomaly, as the delay and improper management of this potential innocuous condition could be disastrous.

## Conclusion

The delay in referring this child to us and the use of gentamycin eye drop for a period of 6 days at the referral clinic is a pointer to poor knowledge about this disorder. We advocate for an improvement in the awareness of this disorder among healthcare professionals both in public and private facilities particularly among those who are involved in obstetric and neonatal care.
